# Primary Ciliary Dyskinesia

**DOI:** 10.1016/j.chpulm.2023.100004

**Published:** 2023-04-05

**Authors:** Michael Glenn O’Connor, Ricardo Mosquera, Hilda Metjian, Meghan Marmor, Kenneth N. Olivier, Adam J. Shapiro

**Affiliations:** a Division of Pulmonary Medicine, Department of Pediatrics, Vanderbilt University Medical Center, Nashville, TN; b Division Pulmonary Medicine, Department of Pediatrics, McGovern Medical School at The University of Texas Health Science Center at Houston and Children’s Memorial Hermann Hospital, Houston, TX; c Division of Pulmonary, Critical Care and Sleep Medicine, National Jewish Health, Denver, CO; d Division of Pulmonary, Allergy and Critical Care Medicine, Stanford University School of Medicine, Stanford, CA; e Division of Pulmonary Diseases and Critical Care Medicine, University of North Carolina at Chapel Hill, Chapel Hill, NC; f Division of Pediatric Respiratory Medicine, McGill University Health Centre Research Institute, Montreal, QC, Canada

**Keywords:** bronchiectasis, Kartagener, laterality, primary ciliary dyskinesia, sinusitis

## Abstract

Primary ciliary dyskinesia (PCD) is a rare but underdiagnosed disorder that affects motile cilia function throughout the body. With increasing prevalence through ongoing genetic discovery, PCD underlies the disease process in a significant number of patients with chronic suppurative lung disease and bronchiectasis when properly investigated using current diagnostic standards. Classic PCD symptoms include chronic rhinosinusitis and otitis, organ laterality defects, infertility, year-round productive cough, and recurrent pneumonias with bronchiectasis. Clinical symptoms of PCD manifest very early in life (often at birth), although diagnosis frequently is delayed because of poor phenotypic recognition and limited access to specialized diagnostic testing. In the past decade, PCD research networks have established specific PCD phenotypes to increase clinical recognition, and the availability of PCD genetic panels in various commercial laboratories has expanded access to an accurate PCD diagnosis greatly. Clinical practice guidelines also were created to guide diagnosis and management of this rare but increasingly recognized suppurative respiratory disease. PCD is more common than previously thought and can be recognized through specific clinical phenotypes in both children and adults. Diagnostic PCD testing outside of highly specialized centers can be difficult, but increased availability of nasal nitric oxide measurement and commercial genetic panels now allows for noninvasive screening and definitive diagnosis regardless of center expertise. Identification of patients with accurately diagnosed PCD is needed worldwide to populate future clinical trials and to develop disease-specific therapies for PCD.

Primary ciliary dyskinesia (PCD) is a rare genetic disease affecting motile cilia throughout the body. The resulting ciliary dysfunction causes the common PCD symptoms of persistent rhinosinusitis and otitis, neonatal respiratory distress, chronic wet cough, recurrent lower respiratory tract infections leading to bronchiectasis, infertility, and organ laterality defects.^1^ Over the past 2 decades, advances in genetic sequencing have expanded the understanding of the origins of PCD. Implementation of multigene panels by numerous commercial genetic testing companies has advanced genetic analysis to the forefront of clinical PCD diagnostics, allowing for definitive PCD diagnosis at any center, regardless of PCD expertise. This review focuses on clinical recognition, accurate diagnosis, and standard therapies that can be used by clinicians who may encounter this rare but increasingly recognized disease.

## Literature Search

For this review, two authors (M. G. O., A. J. S.) queried the PubMed database for articles relating to the search terms *primary ciliary dyskinesia* or *Kartagener*, considering only publications after 2001, when the first gene causing PCD was discovered and the genomic era of PCD investigation began. Results were filtered to exclude cases in nonhumans and case reports, resulting in 562 publications for which abstracts were reviewed. Reviewers next selected publications with clinical impact for full-text evaluation. Additional references were supplemented from authors’ pre-existing literature collections, as warranted.

## Evidence Review

### Epidemiologic Characteristics

The prevalence of PCD is estimated at 1:7,500 to 1:15,000 individuals and occurs across all races and ethnicities.^2^^,^^3^ With these estimates, 25,000 to 50,000 people in North America should be affected with PCD, yet the PCD Foundation (the primary PCD patient advocacy group in North America) has estimated that only 1,000 individuals in North America have received an accurate diagnosis. This low prevalence stems from poor clinical recognition of PCD phenotypes and a lack of easily accessible PCD diagnostic tools outside of highly specialized centers. As a result, the average age at diagnosis is approximately 5 years in some cohorts, but PCD frequently is diagnosed later in childhood or in adulthood.^4^ Unfortunately, a delayed PCD diagnosis is associated with poor respiratory outcomes.^5^ Significant numbers of patients with PCD also are found in adult bronchiectasis cohorts through exome sequencing, revealing that up to 12.5% of these patients have genetically confirmed PCD.^6^

### Pathophysiologic Characteristics

The central biologic pathology in PCD is a lack of effective ciliary movement at the upper and lower airway surfaces. This leads to loss of normal mucociliary clearance with mucostasis and subsequent infection with chronic inflammation.^7^ Abnormal ciliary movement in the paranasal sinuses causes chronic rhinitis and acute on chronic sinusitis, whereas stasis in the lower airways results in chronic wet cough, recurrent pneumonia or bronchitis, and eventual development of bronchiectasis.^8^ Ciliary function also is essential for middle-ear fluid egression, and when abnormal, contributes to frequent otitis media, persistent middle-ear effusions, and hearing loss.^9^ Dysfunction of motile, ependymal cilia in the CNS may result in hydrocephalus, although only a small portion of patients with PCD with specific genetic defects actually demonstrate this.^10^^,^^11^ Finally, ciliary movement directs the embryonic nodal system responsible for proper organ laterality placement in utero and for propulsive structures in sperm tails and fallopian fimbriae of the reproductive system.^12^^,^^13^

### Disease Phenotypes

Chronic cough and rhinorrhea are common symptoms in outpatient pulmonary clinics. Although PCD is rarely the underlying cause of these symptoms, recognizing the PCD-specific clinical aspects of these affords clinicians the proper index of suspicion to detect PCD cases. Research from the Genetic Disorders of Mucociliary Clearance Consortium and International PCD cohorts has defined the key clinical features of PCD in children, including: (1) year-round wet or productive cough that begins in early infancy, (2) year-round rhinitis that also begins in early infancy, (3) unexplained neonatal respiratory distress despite term birth, and (4) presence of an organ laterality defect (ie, situs inversus totalis or situs ambiguus).^14^ Adult PCD cohorts from the Genetic Disorders of Mucociliary Clearance Consortium also have shown high prevalence of these key clinical symptoms in patients who receive a diagnosis after 18 years of age,^15^ with the evolution of chronic rhinitis to chronic sinusitis (Table 1).

**Table 1:** Key Clinical Symptoms of PCD in Adults and Children

When to Suspect PCD in Pediatric and Adult Patients
Symptoms highly suggestive of PCD in children • At least two of four key clinical symptoms^14^ ◦ Year-round wet cough with onset before 6 mo of age or ◦ Year-round nasal congestion with onset before 6 mo of age or ◦ Neonatal respiratory distress at term birth requiring supplemental oxygen or positive pressure support for at least 24 h or ◦ An organ laterality defect • Unexplained bronchiectasis^a^ with chronic oto-sino-pulmonary disease
Symptoms highly suggestive of PCD in adults: • At least two of four key clinical symptoms above^14^ or • Unexplained bronchiectasis^a^ with chronic rhinosinusitis or • Unexplained bronchiectasis^a^ with ongoing otitis in adulthood or • Unexplained bronchiectasis^a^ with an organ laterality defect or • Unexplained bronchiectasis^a^ with male or female infertility or • Unexplained bronchiectasis^a^ with chronic respiratory symptoms since early childhood

PCD = primary ciliary dyskinesia.

a Bronchiectasis in PCD shows a predominance for middle- and lower-lobe involvement.

Approximately 80% of children with PCD have a history of neonatal respiratory distress at term birth,^14^^,^^16^ but this prevalence decreases for those who receive a diagnosis in adulthood, likely because of recall bias. In PCD, the onset of neonatal respiratory distress often is delayed for 12 to 24 h, as opposed to other causes of neonatal respiratory distress (ie, neonatal pneumonia, meconium aspiration, and transient tachypnea of the newborn), which appear immediately after birth.^16^ The respiratory distress in infants with PCD often is prolonged, requiring an average of 2 weeks of respiratory support or supplemental oxygen, and often is accompanied by persistent upper lobe or shifting lobar atelectasis on neonatal chest radiography.^16^^,^^17^

The chronic cough in PCD is almost always wet, and even infants are sometimes able to expectorate sputum, which is rare in other pediatric respiratory conditions. The characteristic wet cough never resolves completely, even after prolonged antibiotic therapy. The chronic rhinitis of PCD is similarly fastidious, never completely resolving even with aggressive antibiotic and topical nasal therapy. In classic PCD, both the wet cough and nasal congestion appear before 6 months of age (often immediately after birth); later onset of these symptoms (ie, during adolescence or adulthood) is not characteristic of PCD and should push clinicians toward other possible diagnoses.

Most patients with PCD also have recurrent otitis media and persistent middle-ear effusions in childhood. For many, these are accompanied by hearing deficits and language delay.^9^^,^^18^ Repeat myringotomy tube placement features prominently in patient histories. A complete lack of recurrent otitis or ear effusions is exceedingly rare in PCD. For unknown reasons, ear disease greatly improves in many adolescent patients with PCD, yet some adults with PCD continue to experience otitis media and hearing deficits.^19^

Recurrent lower respiratory tract infections are also a major clinical finding in PCD, and the persistent, suppurative lung inflammation behind these infections eventually leads to development of bronchiectasis. Approximately 50% of children demonstrate bronchiectasis by 8 years of age, and nearly 100% of adults with PCD display bronchiectasis, often with a predilection for middle- and lower-lobe involvement.^20^^,^^21^

Other common PCD features found mainly in adulthood include chronic sinusitis and infertility. Most adult patients with PCD require endoscopic sinus surgery for chronic sinus disease and may have hypoplastic or absent frontal and sphenoid sinuses, although their burden of nasal polyposis seems relatively low compared with that of other suppurative diseases, like cystic fibrosis.^22^, ^23^, ^24^ Most men with PCD reportedly are infertile because of sperm immotility (because sperm tail propulsion relies on the same motile ciliary apparatus as respiratory cilia), and intracytoplasmic sperm injection is required for successful conception.^13^ Women with PCD can show reduced fertility secondary to dysfunction of oviductal fimbriae motion, yet a proportion can still bear children without fertility assistance.^25^ The prevalence of ectopic (tubal) pregnancy also may be increased in women with PCD.^26^

Because of dysfunction of nodal cilia in developing embryos, organ laterality defects frequently occur in PCD, with slightly < 50% of patients displaying mirror-image inversion of all internal organs (situs inversus totalis). However, closer inspection of cardiac, thoracic, and abdominal organs sometimes reveals other laterality abnormalities in 12% to 20% of patients, known as *situs ambiguus*. In situs ambiguus, defects may arise as grouped patterns (isomerism) or in isolation along a spectrum between normal organ placement and situs inversus totalis. Cardiac defects may include isolated dextrocardia, simple septal defects, or complex congenital heart disease (heterotaxy), whereas thoracic defects can include bilateral bilobed or trilobed lungs and abdominal defects may comprise dextrogastria, polysplenia or asplenia, interrupted inferior vena cava, or other alterations (Table 2).^27^ Thus, the presence of any organ laterality defect, in isolation or throughout the body, should greatly increase the suspicion of PCD in patients with chronic respiratory disease. Notably, a lack of organ laterality defects does not rule out PCD because these occur randomly, even within related patients affected by identical PCD gene variants. People with disease-causing variants in genes encoding radial spoke or central apparatus components do not manifest organ laterality defects because these structures are not necessary for proper function of embryonic nodal cilia and normal organ placement.

**Table 2:** Examples of Possible Organ Laterality Defects With Situs Ambiguus in PCD

Cardiac defects • Isolated dextrocardia • Simple congenital heart defects (ASD, VSD, and so forth) • Complex congenital heart defects (heterotaxy) • Atrial isomerism • Common atrium • Atrioventricular discordance • Ventriculoarterial discordance • Others	Vascular defects • Right aortic arch • Bilateral or left superior vena cava • Interrupted inferior vena cava • Levotransposition or dextrotransposition of the great vessels • Anomalous pulmonary venous return • Others
Abdominal defects • Situs inversus abdominalis • Midline liver • Dextrogastria • Polysplenia or asplenia (right or left sided) • Intestinal malrotation • Horseshoe kidney • Annular pancreas • Duodenal atresia • Fused adrenal glands • Extrahepatic biliary atresia • Others	Pulmonary defects • Left pulmonary isomerism (bilateral bilobed lungs or hyparterial bronchi) • Right pulmonary isomerism (bilateral trilobed lungs or eparterial bronchi) • Pulmonary situs inversus

Any defect may occur in isolation or in combination with other defects across the cardiac, vascular, pulmonary, or abdominal compartments. Specific patterns of left or right isomerism are possible with primary ciliary dyskinesia, but identification of an isolated lesion also should raise suspicion greatly of primary ciliary dyskinesia in a patient with chronic respiratory, sinonasal, or ear disease.^28^ ASD = atrial septal defect; VSD = ventricular septal defect.

According to American Thoracic Society clinical practice guidelines, the presence of at least two key clinical symptoms in a patient should prompt further PCD diagnostic testing.^28^ Moreover, a diagnosis of PCD should be pursued in adults with bronchiectasis accompanied by chronic sinonasal disease, infertility, an organ laterality defect, or the onset of chronic respiratory symptoms in early childhood (Table 3).

**Table 3:** Summary of Available PCD Diagnostic Testing

Testing	Testing **P**opulation and **S**pecific **C**onsiderations	Positive **R**esults	Comments
nNO measurement	• Cystic fibrosis must be ruled out • Must be ≥ 5 y of age and able to exhale against a resistor^a^ • In North America, only available in accredited PCD Foundation centers with chemiluminescence analyzers and approved protocols^29^ • Must be in baseline state of health for at least 2 wk	< 77 nL/min by exhalation against resistor maneuver on at least two separate occasions^29^	• Increasing false-negative results with specific PCD genotypes (*RSPH1*, *CCDC103*, and so forth)^30^^,^^31^ • False-positive results are possible (eg, acute viral illness, cystic fibrosis, epistaxis, rare forms of immunodeficiency)^32^^,^^33^ • Repeat testing is required
Genetic testing	• Perform at any age and anytime • Highly feasible in most clinical care centers • Most commercial multigene panels evaluate ≥ 40 known PCD genes through NGS, including deletion and duplication analysis • Whole exome sequencing will increase sensitivity, but is not covered by most insurance	Two pathogenic or likely pathogenic variants within one autosomal recessive PCD-causing gene^31^^b^	• Testing is nondiagnostic in 20-30% of cases • Single variants in different PCD genes are nondiagnostic, because both variants must occur within the same gene to cause PCD • Frequently results in VUS, which are nondiagnostic • Different PCD genes included in various commercial panels will affect overall sensitivity • Known intronic variants in *DNAH11* and *CCDC39* will not be detected on NGS panels^31^
TEM of respiratory cilia	• Perform at any age • Must be in baseline state of health for at least 2 wk • Requires a pathology laboratory with extensive experience in ciliary TEM processing and analysis to avoid insufficient sampling	Class 1 ultrastructural defects: (1) outer dynein arm defect, (2) outer and inner dynein arm defect, (3) absent inner dynein arm with microtubule disorganization^34^	• 30% of the patients with PCD harbor normal or nondiagnostic ciliary ultrastructure^35^ • Changes aside from class 1 defects are nondiagnostic^34^ • Not applicable to certain ciliary production defects caused by specific genes (*CCNO*, *MCIDAS*)^31^
High-speed video microscopy with beat pattern analysis	• Perform at any age • Must be in baseline state of health for at least 2 wk • Limited resources and expertise for this in North America • Requires repeat analysis on three separate visits or after air-liquid cellular regrowth to avoid secondary causes of dyskinesia^36^	Abnormal ciliary beat pattern	• Normal cilia waveform analysis does not rule out a PCD diagnosis, because some mutations may result in normal or near-normal movement • Beat-pattern interpretation is nonstandardized and may differ between reviewers and laboratories^36^ • Abnormal ciliary beat pattern also may result from a secondary cause, like acute respiratory illness
Immunofluorescence testing of ciliary proteins	• Perform at any age • Must be in baseline state of health for at least 2 wk • Limited resources and expertise for this in North America	Absent fluorescence of ciliary proteins in specific axonemal components	• False-negative results in specific genetic forms of PCD, including cases caused by *DNAH11* and *HYDIN* variants • Not applicable to certain ciliary production defects caused by specific genes (*CCNO*, *MCIDAS*)^31^

NGS = next generation sequencing; nNO = nasal nitric oxide; PCD = primary ciliary dyskinesia; TEM = transmission electron microscopy; VUS = variant of uncertain significance.

a Nasal nitric oxide measurement can be performed down to 2 years of age using a tidal breathing technique, but diagnostic cutoff values are not well defined for this group.

b Variants should be proven biallelic with phase testing of other family members as possible. In *RPGR*, *OFD1*, *PIH1D3* (X-linked), and *FOXJ1* (autosomal dominant), only one pathogenic or likely pathogenic variant is required for a PCD diagnosis.

### Diagnosis

After establishing an appropriate PCD clinical phenotype, diagnostic testing can be performed with a myriad of tests. No single test is 100% sensitive or specific for a PCD diagnosis, and each test has limitations related to availability and feasibility outside of highly specialized centers. In North America, PCD diagnosis rests on screening nasal nitric oxide (nNO) measurement and confirmatory genetic panel testing, followed by ciliary transmission electron microscopy (TEM) as needed for inconclusive genetic results.^28^ Some highly experienced European centers complement this approach with high-speed videomicroscopy analysis of ciliary beat pattern and ciliary protein immunofluorescence, neither of which are available routinely in North America.^36^ For individuals 5 years of age and older, American Thoracic Society guidelines recommend nNO measurement as the first-line diagnostic test when available.^28^ However, nNO testing needs to be performed in an accredited PCD Foundation center with a chemiluminescence nitric oxide analyzer using an approved measurement protocol.^29^ In patients younger than 5 years or without access to nNO chemiluminescence testing, the recommended first-line test is PCD genetic panel analysis.

nNO testing has high diagnostic accuracy for PCD diagnosed by genetics or TEM.^37^ The test is noninvasive, can be performed quickly, and is inexpensive, and results are available immediately.^29^^,^^37^ The biologic mechanism affecting nNO levels in PCD is not understood fully, and investigations have shown that low nNO values do not result from decreased nitric oxide (NO) production, NO synthase deficits, increased NO breakdown, mucus obstruction of sinonasal passages, or colonization of nasal secretions with denitrifying bacteria.^38^ It is possible that expression or activity of a NO metabolic protein downstream of NO synthase is regulated abnormally in association with ciliary dysfunction, but this remains unproven.^39^

It has been observed robustly that 90% to 95% of patients with PCD will show repeatedly low nNO values when using a sensitive and specific cutoff value of < 77 nL/min.^40^ Rare, but increasing, cases that often are associated with specific PCD genotypes (eg, *RSPH1*, *CCDC103*, and *CFAP221*) sometimes result in nNO values of more than the 77-nL/min cutoff.^27^^,^^30^^,^^41^^,^^42^ These cases represent a minority, but important, proportion of currently recognized PCD cases, and the use of a higher cutoff value (108 nL/min) for PCD screening has been suggested.^41^ Consequently, when the clinical suspicion for PCD is high, nNO values of > 77 nL/min should not rule out PCD, and additional testing with genetics and TEM is still warranted. Most individuals with PCD maintain reduced nNO levels for life, and normalization of nNO levels should alert clinicians to an alternative diagnosis.

**Figure 1:**
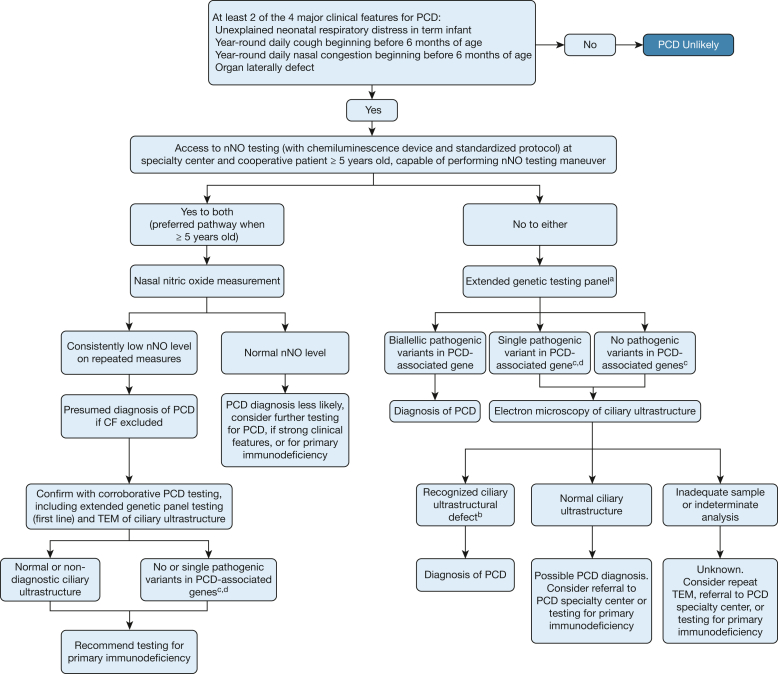
American Thoracic Society clinical practice guideline algorithm for diagnosing PCD. ^a^Genetic panels testing for mutations in > 12 diseases associated with PCD genes, including deletion and duplication analysis. ^b^Known disease-associated TEM-identified ultrastructural defects include outer dynein arm defects, outer dynein arm plus inner dynein arm defects, and inner dynein arm defects with microtubular disorganization. ^c^In genes associated with autosomal recessive trait. ^d^Or presence of variants of unknown significance. CF = cystic fibrosis; nNO = nasal nitric oxide; PCD = primary ciliary dyskinesia; TEM = transmission electron microscopy. (Reprinted with permission of the American Thoracic Society. Copyright © 2023 American Thoracic Society. All rights reserved.^28^^,^^45^)

Limitations of nNO testing include false-positive results in patients with cystic fibrosis or with recent viral illness, epistaxis, or sinonasal instrumentation (Table 3).^41^^,^^43^ In addition, evidence is increasing that patients with rare immunodeficiencies can have repeatedly low nNO values.^32^^,^^44^ Specific evidence exists for repeatedly low nNO values with *RAG1* and *PIK3CD* genetic variants, but it is possible that other immunodeficiencies with low nNO values have yet to be described.^32^^,^^33^ When immunodeficiencies are misdiagnosed as PCD based on nNO test results alone, patients risk significant morbidity and even mortality secondary to untreated immune issues.^30^ For these reasons, updated American Thoracic Society guidelines recommend all cases of PCD be verified through genetic or TEM analysis, and when these tests are inconclusive, consultation with an immunology specialist is necessary (Fig 1).^45^

Genetic panel testing with next generation sequencing is rapidly becoming the de facto first-line test for PCD across North America because of the high feasibility of this testing method regardless of expertise in PCD. Commercially available genetic panel tests require only a sample of blood or buccal cells, which can be collected in medical resource-poor locations. However, the intricacies of PCD genetics often pose issues for clinicians interpreting these results. More than 50 PCD genes have been described, and most follow an autosomal recessive pattern of inheritance.^8^^,^^31^ Importantly, no reports exist of digenic variants causing PCD, and thus a genetic diagnosis of PCD is made by the presence of two pathogenic or likely pathogenic variants (per American College of Genetics and Genomics pathogenicity classification) within the same gene (Table 3).^1^^,^^46^ Single variants in different PCD-causing genes are not diagnostic of PCD. Furthermore, a diagnosis cannot be confirmed when one variant is pathogenic but the other is a variant of uncertain significance, even when the variants have been confirmed to be on opposite chromosomes. Current next generation sequencing genetic panel testing does not provide a confirmatory diagnosis in 20% to 30% of patients with PCD, but additional PCD-causing genes are being identified continually, and new genes frequently are being added to existing commercial panels.^31^ As clinical exome sequencing becomes more widely available, one can expect the proportion of genetically diagnosed cases of PCD to increase further, as has been shown in research protocols.^47^ Consultation with a clinical geneticist often is required to interpret PCD genetic testing results correctly.

TEM analysis of respiratory cilia is now a second-line PCD diagnostic test because numerous technical challenges prevent successful TEM studies outside of highly experienced pathology laboratories. These include difficulty obtaining an adequate biopsy sample and appropriately processing the sample to obtain a minimum of 50 unique ciliary cross sections for interpretation.^48^ Although ciliated cells can be obtained nasally in nonsedated, cooperative patients, inexperienced operators may collect samples when patients are not at a baseline state of health or may fail to probe deep enough under the inferior turbinate, making cellular yields too low for reliable TEM analysis. These challenges lead to sample failure in 12% to 40% of patients who undergo TEM,^49^^,^^48^ even at experienced centers. Even when these critical steps are achieved successfully, approximately 30% of individuals with PCD will show normal ciliary ultrastructure on TEM.^35^^,^^50^^,^^51^ According to European Respiratory Society consensus, only class 1 defects (absent outer dynein arms, absent outer and inner dynein arms, or absent inner dynein arms with microtubule disorganization) are diagnostic of PCD, whereas the remainder of TEM changes often are secondary to processing artifact or inflammation and are only suggestive, rather than diagnostic, of a possible ultrastructural defect.^34^

### Clinical Monitoring

Making a diagnosis early in life is an essential step to prescribing effective therapies for individuals with PCD.^4^^,^^52^ When a diagnosis of PCD is confirmed later in life or after serious lung damage has occurred already, treatment effectiveness may be impaired. Long-term outcomes in PCD are becoming more apparent as cohorts with a proper diagnosis are followed up through childhood and into adulthood. Severe pulmonary function impairment, supplemental oxygen dependence, or need for lung transplantation are reported in 38% to 51% of adults with PCD.^21^^,^^53^ Overall mortality and life expectancy and outcomes after lung transplantation have been explored poorly in patients with PCD.^54^ With > 50 distinct genes causing PCD, clinical outcomes are highly variable, but diagnosis of specific PCD genotypes leads to better understanding of clinical variability and disease progression. As an example, individuals with PCD resulting from absent inner dynein arms with microtubule disorganization (or corresponding variants in the *CCDC39* or *CCDC40* genes) experience worse respiratory and nutritional outcomes,^55^ whereas milder respiratory outcomes have been seen with the *RSPH1* genotype.^27^ Continued observation and evidence are needed to understand new genotype-phenotype relationships and to evaluate if prior observations remain true.

The PCD Foundation created a consensus statement for disease monitoring and therapies^1^ and suggests that at the time of PCD diagnosis, evaluation for organ laterality abnormalities be considered. Approximately one-half of individuals with PCD have an organ laterality abnormality,^27^ which may include the absence of a functioning spleen compromising the immune system.^56^ Verification of normal splenic function is especially important because it can predispose patients to life-threatening infections and may necessitate accelerated vaccinations and antibiotic prophylaxis.^57^ Approximately 10% of individuals with PCD also have congenital heart disease, and thus screening with echocardiography is suggested at the time of PCD diagnosis.^58^

Patients with a diagnosis of PCD should be seen two to four times annually by a pulmonologist experienced in the management of chronic suppurative lung disease or at an accredited PCD Foundation center.^1^ During these visits, lung function testing and bacterial respiratory culture analyses are recommended. Respiratory culture analyses for nontuberculous mycobacteria also are recommended at least every 2 years, although this may be performed more frequently in patients receiving chronic azithromycin therapy. In contrast to individuals with cystic fibrosis, where *Pseudomonas aeruginosa* and *Staphylococcus aureus* are the most common bacterial respiratory pathogens, *Haemophilus influenzae* is the most commonly isolated respiratory pathogen in children and adolescents with PCD, whereas *P aeruginosa* appears in 20% to 40% of pediatric respiratory cultures. The prevalence of *P aeruginosa* increases in adults with PCD, becoming even more prevalent after 30 years of age. Mucoid *P aeruginosa* in patients with PCD is comparatively less prevalent than in patients with cystic fibrosis, but also increases in older adults.^59^ Allergic bronchopulmonary aspergillosis has been reported in pediatric and adult patients with PCD, but this entity overall seems less common than in patients with cystic fibrosis.^60^^,^^61^

Current guidelines recommend obtaining chest CT scan imaging for evaluation of bronchiectasis, after the diagnosis of PCD has been confirmed.^1^ Visualization of the lung parenchyma with careful attention to the development and progression of bronchiectasis is an important part of PCD disease monitoring; the appropriate interval for repeat CT scan imaging is not known.

Although clinical variability of ear and sinus manifestations exists in individuals with PCD, initial consultation and establishing care with an otolaryngology expert is an important part of comprehensive care in this population.^62^ From an otic perspective, routine hearing monitoring early in life can improve hearing loss, can prevent delay in speech development, and can improve school performance.^63^ Treatment of hearing loss also is an important quality of life issue for both adult and pediatric patients with PCD.^19^ Sinus involvement with or without polyposis can be quite cumbersome in PCD, and this significantly affects quality of life, often worsening as patients age.^62^ However, the ideal time for medical vs surgical intervention is unique to each patient and involves continued monitoring by rhinology specialists in otolaryngology clinics.

### Therapeutics

Treatment primarily consists of regular airway clearance routines, management of airway inflammation, and treatment of acute pulmonary infections.^1^ Randomized controlled trials in PCD patient populations are limited; however, some initial studies support available treatments (Table 4).^1^^,^^56^

**Table 4:** Clinical Monitoring and Therapies for PCD

Method	Description
Recommended clinical monitoring^1^	Pulmonary • Outpatient visits 2-4 times/y with spirometry testing • Airway microbiology culture 2-4 times/y • NonTB mycobacteria culture every 2 y and with unexpected lung function decline (and more frequently if receiving azithromycin therapy) • Chest radiography every 2-4 y or with illness • Chest CT scan imaging at least once at or after diagnosis • Allergic bronchopulmonary aspergillosis testing with unexpected lung function decline Otolaryngology • Outpatient visits 1-2 times/y in children, as needed in adults • Audiology evaluation at diagnosis and as needed Organ laterality • Echocardiography at diagnosis^56^ • Abdominal imaging at diagnosis (usually with ultrasound, but can be visualized on CT scan; confirmation of spleen presence and consider testing for splenic function when splenic anomalies present)^56^
Recommended therapies in all patients^1^	Pulmonary • Airway clearance at least daily (more frequent with respiratory illness) • Cardiovascular exercise at least daily • Azithromycin thrice weekly when frequent respiratory exacerbations occur^64^ • Inhaled antibiotics for eradication of new *P aeruginosa* respiratory culture • 14-21 d of oral or parenteral antibiotics for acute respiratory exacerbations • Standard vaccinations, including annual influenza vaccine, extended serotype pneumococcal vaccine Otolaryngology • Nasal sinus lavage: daily when appropriate
Treatments used on a case-by-case basis^1^	• Inhaled 3%-7% hypertonic saline at least daily (more frequent with respiratory illness)^65^, ^66^, ^67^ • Inhaled DNase (dornase alfa): long-term use may worsen pulmonary function and increase exacerbations in bronchiectasis^67^^,^^68^ • Chronic inhaled antibiotics for *P aeruginosa* sputum colonization
Treatments not routinely recommended^1^	• Inhaled corticosteroids (outside of asthma diagnosis) • IV immunoglobulin (outside of diagnosed immunodeficiency) • Regular bronchodilators (outside of asthma or premedication for inhaled agents) • Lobectomy^69^

PCD = primary ciliary dyskinesia.

The mainstay of PCD care is to restore effective airway clearance. Because the mucus in patients with PCD is much less dehydrated with lower percent solids than in cystic fibrosis,^70^ cough clearance is relatively preserved and, when stimulated, can clear lower airway secretions. This can be accomplished through regular use of handheld positive expiratory pressure devices, intrapulmonary percussive ventilation devices, oscillating chest wall vests, or various breathing techniques.^71^ A short-term comparison of chest percussion and postural drainage with high-frequency chest wall oscillation therapy in patients with PCD found no difference in pulmonary function test results, with both groups showing significantly improved pulmonary function.^72^ Aerobic exercise also is an important part of PCD clinical treatment. In some centers, clearance of airway mucus in patients with PCD is performed with dedicated aerobic exercise of ≥ 30 min coupled with huff coughing.^73^ To be an effective means of airway clearance, aerobic exercise needs to be performed consistently.

In addition to standard airway clearance, a commonly used regimen for patients with PCD is daily inhalation of 3% to 7% nebulized hypertonic saline.^1^ Although the use of inhaled hypertonic saline has not been well studied in PCD, its ability to induce a cough reflex has led to its clinical adoption by many PCD caregivers.^66^ One underpowered study examining the efficacy of inhaled hypertonic saline found no significant differences in respiratory exacerbations, lung function, or quality of life on the St. George’s Respiratory Questionnaire score when compared with inhaled isotonic saline. However, the health perception score on the Quality of Life Questionnaire-Bronchiectasis instrument did improve significantly with inhaled hypertonic saline.^65^

In 2020, results from the first randomized, placebo-controlled clinical trial in patients with PCD demonstrated clinical efficacy for maintenance dosing of azithromycin.^64^ It was found to be well tolerated and to reduce pulmonary exacerbations by nearly one-half without increasing antibiotic resistance of sputum organisms.

Inhaled DNase (dornase alfa) has not been studied specifically in individuals with PCD, but its use in patients with bronchiectasis without cystic fibrosis showed no clinical benefits and even worsened outcomes (lung function and respiratory exacerbations) in some patients.^1^^,^^67^^,^^68^ Thus, DNase is used on a case-by-case basis in patients with PCD,^1^ but providers must be aware of its potentially harmful effects. Bronchodilators frequently are used as part of an airway clearance routine and as pretreatment before inhaled hypertonic saline, but no evidence exists of long-term benefit from routine use of bronchodilators.^74^ In addition, no evidence exists for the routine use of inhaled corticosteroids outside of those patients with PCD with a concurrent asthma diagnosis.^75^ It is rare for a patient with PCD to have a concurrent immunodeficiency, and thus routine treatment with immunoglobulin replacement is not recommended.^76^ Lobectomy similarly is not recommended in PCD because it is associated with poor outcomes and will not halt disease progression (Table 4).^69^

Broad spectrum oral antibiotics should be used for mild, acute respiratory exacerbations, which most often present with increased cough, sputum, work of breathing, and sinonasal congestion (above the baseline state) for at least 5 to 7 days in patients with PCD. Antibiotics should target recent organisms isolated from surveillance sputum cultures. Moderate or severe exacerbations should be treated with parenteral antibiotics. As in other suppurative respiratory diseases, longer therapeutic courses (14-21 days) are used commonly, although no evidence supports this. Acutely increasing the frequency of airway clearance sessions and inhaled mucolytics (hypertonic saline and airway clearance up to qid) also may be performed during acute exacerbations. Inhaled antibiotics have not been studied in patients with PCD, and their use largely has been reserved for eradication or maintenance therapy in patients with sputum cultures showing positive results for *P aeruginosa*.

## Closing Section

Although the next decade of PCD research will continue to expand genetic discovery and genotype-phenotype correlations, a notable shift in therapeutic studies is expected. These should include multiple randomized clinical trials assessing efficacy of repurposed therapies from suppurative respiratory disease as well as novel, disease-specific treatments for PCD. A phase 2 trial assessing mucus hydration therapies in PCD recently completed enrollment, and various entities are evaluating inhaled messenger RNA therapies for protein-specific defect correction in PCD.^77^ Multicenter observational protocols also are enrolling patients for cross-sectional assessment of upper airway disease in PCD,^78^^,^^79^ while other studies are investigating parameters of natural disease progression in adult populations with PCD.^80^ Ongoing patient registries through the PCD Foundation in North America and the International PCD Cohort in Europe are vital resources to support these efforts, which ultimately will result in effective therapies and improved patient outcomes in this rare but increasingly recognized respiratory disease.

## Summary

PCD is underdiagnosed worldwide, but knowledge of specific clinical phenotypes, coupled with new and accessible diagnostic testing, is allowing increased detection of this chronic respiratory disease. Although much of the monitoring and therapy remains unproven in PCD, regular clinical follow-up, daily airway clearance, reduction of chronic airway inflammation, and aggressive treatment of upper and lower respiratory tract infections are the cornerstones of care for patients with PCD. Accurate PCD diagnosis to grow patient cohorts is the best way to increase public awareness for this rare disease and to populate future clinical trials.

## Funding/Support

Funding support for research was provided to K. N. O. and A. J. S. by the 10.13039/100000050National Heart, Lung, and Blood Institute (NHLBI), 10.13039/100000002National Institutes of Health [Grant U54HL096458]. The Genetic Disorders of Mucociliary Clearance Consortium [Grant U54HL096458] is part of the National Center for Advancing Translational Sciences (NCATS) Rare Diseases Clinical Research Network (RDCRN) and supported by the RDCRN Data Management and Coordinating Center [Grant U2CTR002818]. RDCRN is an initiative of the Office of Rare Diseases Research funded through a collaboration between NCATS and NHLBI.

## Financial/Nonfinancial Disclosures

None declared.
